# Mining for Candidate Genes Controlling Secondary Growth of the Carrot Storage Root

**DOI:** 10.3390/ijms21124263

**Published:** 2020-06-15

**Authors:** Alicja Macko-Podgórni, Katarzyna Stelmach, Kornelia Kwolek, Gabriela Machaj, Shelby Ellison, Douglas A. Senalik, Philipp W. Simon, Dariusz Grzebelus

**Affiliations:** 1 Department of Plant Biology and Biotechnology, University of Agriculture in Krakow, 31-120 Krakow, Poland; k.stelmach@student.urk.edu.pl (K.S.); k.kwolek@student.urk.edu.pl (K.K.); g.machaj@student.urk.edu.pl (G.M.); 2 Department of Horticulture, University of Wisconsin-Madison, Madison, WI 53706, USA; slrepinski@wisc.edu; 3 USDA ARS Vegetable Crops Research Unit, University of Wisconsin-Madison, Madison, WI 53706, USA; douglas.senalik@usda.gov (D.A.S.); philipp.simon@usda.gov (P.W.S.)

**Keywords:** association mapping, *Daucus carota*, *DCAF1*, *BTAF1*, root shape, storage root

## Abstract

Background: Diverse groups of carrot cultivars have been developed to meet consumer demands and industry needs. Varietal groups of the cultivated carrot are defined based on the shape of roots. However, little is known about the genetic basis of root shape determination. Methods: Here, we used 307 carrot plants from 103 open-pollinated cultivars for a genome wide association study to identify genomic regions associated with the storage root morphology. Results: A 180 kb-long region on carrot chromosome 1 explained 10% of the total observed phenotypic variance in the shoulder diameter. Within that region, *DcDCAF1* and *DcBTAF1* genes were proposed as candidates controlling secondary growth of the carrot storage root. Their expression profiles differed between the cultivated and the wild carrots, likely indicating that their elevated expression was required for the development of edible roots. They also showed higher expression at the secondary root growth stage in cultivars producing thick roots, as compared to those developing thin roots. Conclusions: We provided evidence for a likely involvement of *DcDCAF1* and/or *DcBTAF1* in the development of the carrot storage root and developed a genotyping assay facilitating the identification of variants in the region on carrot chromosome 1 associated with secondary growth of the carrot root.

## 1. Introduction

Carrot is widely used as a fresh or processing vegetable. It is the most significant source of beta-carotene in the human diet. To meet consumers’ demands and industry needs, a range of cultivar types suitable for fresh market or processing have been developed. Root morphology is one of the major attributes defining these types. Since the 16th century, root traits have been described and become key parameters in breeding programs [1]. However, little is known about the genetic basis of the carrot root shape determination. Possibly, it is determined by many genes with small individual effects and markedly modified by the environment. The impact of environmental factors, e.g., planting density [2], water regime [3], soil forming [4], temperature [5], and nutrient availability [6] on carrot root growth have been described. It was also reported that heterosis resulted in increased carrot storage root biomass and improved its quality [7] and a strong linear relationship between shoot and root biomass was shown [8].

The ability to develop storage roots is the hallmark of all cultivated carrots and a key component of the carrot domestication syndrome [9]. To date, published reports have been focused on genetic determinants governing root traits associated with carrot domestication [10,11]. In particular, *DcAHLc1*, a gene encoding an AT-hook motif nuclear localized protein, possibly engaged in developmental regulation, was shown to be important for the development of the carrot storage root [10,12,13]. 

Research on the identification of genetic factors controlling development of the carrot storage root has suffered from low reliability of phenotyping. A significant improvement in that area has been possible following the use of automated image analysis [8]. However, genomic regions associated with root thickening have not been identified. Recent comparative analysis of transcriptomes of cultivated and wild carrot roots revealed that genes encoding transcription factors, proteins involved in post-translational modifications, cell cycle regulation, vascular strand development, and cellular transport are involved in the formation of the carrot storage root, revealing the complex molecular interactions driving this developmental process [14]. 

With the recent improvement in high-throughput genotyping methods and development of analytical methods for QTL identification, discovery of genomic regions associated with a plant phenotype, including those with minor effects, have become possible by means of genome wide association studies (GWAS). Small effect loci controlling maize leaf architecture [15], root traits in wheat seedlings [16] or panicle number and grain number per panicle in F1 rice varieties [17] have been described. The rapid linkage disequilibrium (LD) decay generally observed in the carrot genome suggests that the GWAS strategy should be applicable for identifying candidate genes [9]. To date, GWAS has been successfully used to identify QTLs governing accumulation of terpenes [18] and carotenoids [11,19] in carrot roots.

Here, we used GWAS to reveal genomic regions involved in the determination of the carrot root shape. We investigated a collection of plants from a range of open-pollinated carrot cultivars producing roots of different shapes, eventually aiming at the identification of single-nucleotide polymorphisms facilitating marker-assisted selection.

## 2. Results

### 2.1. Phenotyping and Genotyping

We used 327 carrot plants for phenotyping and genotyping-by-sequencing (GBS). Individuals representing outlier phenotypes, and those for which the quality of the GBS data was low were excluded and subsequent analyses were performed on 307 plants. A dataset D1 comprised of 1808 high quality SNPs was used to correct for relatedness and genetic structure while dataset D2, containing 75,525 SNPs, was used for GWAS. 

We observed large phenotypic diversity of all four morphological parameters (Table 1, Supplementary Figure S1). Differences were noted not only among the varietal groups but also within those groups (Supplementary Table S1). No significant correlation was observed for the measured parameters (data not shown).

### 2.2. Population Structure

**Figure 1:**
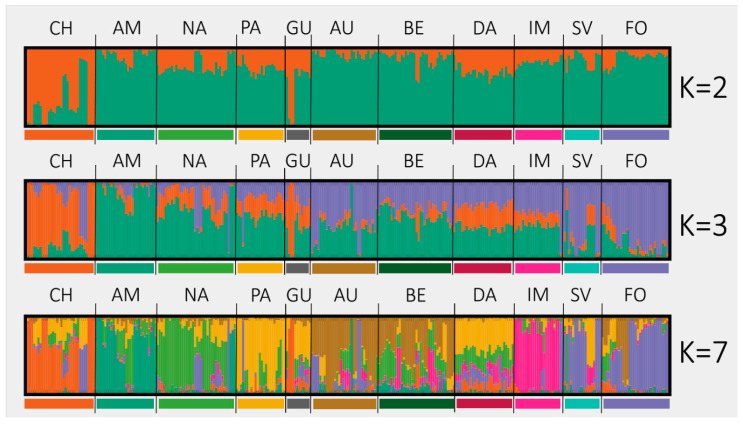
Estimated population structure of the 307 carrot plants for K = 2, K = 3 and K = 7. Each plant is represented by vertical line divided into colored segments representing the membership fractions (Q) in the K clusters. Cultivar types are labeled by different color bars below the graphs and letters above the graphs; CH—Chantenay, AM—Amsterdam, NA—Nantes, PA—Paris Market, GU—Guerande/Oxheart, AU—Autumn King, BE—Berlicum, DA—Danvers, IM—Imperator, SV—St. Valery, FO—fodder carrot.

For D1, the highest ∆K values were observed for K = 2 [∆K(2) = 262.78], K = 3 [∆K(3) = 115.80] and K = 7 [∆K(7) = 70.38]. ∆K values for K = 4–5 and K = 8–11 were relatively low, ranging from 0.32 to 41.98. For two assumed clusters low admixture was observed, whereas for three and seven clusters the level of admixture was 31.27% (96 accessions with membership coefficient (Q) not exceeding 0.5) and 28.66% (88 accessions with Q < 0.5), respectively (Figure 1). 

The assumption of two clusters resulted in clear separation of 28 plants belonging to the Chantenay varietal group, characterized by a conic, stump and thick root. The remaining plants of various root types were grouped into the other cluster. When three clusters were assumed, the previously extracted Chantenay cluster remained unchanged, whereas the remaining plants were divided into two clusters. One comprised plants belonging mostly to Amsterdam, Early Short Horn, and Nantes types, which were quite diverse with regard to the length of the blunt-ended root. Within the other new cluster, Autumn King and St. Valery types, and fodder carrot, characterized by long conical roots, prevailed. 

With seven clusters assumed, further separation of root types was observed. New clusters comprised: Amsterdam type with medium-length cylindrical and stump root (K1); Chantenay type (K2 which remained virtually unchanged); Autumn King and Berlicum types with long conic stump roots (K3); Early Short Horn cultivars with short conical roots (K4); St. Valery type and fodder carrot with long conical and mostly pointed roots (K5); Nantes type with medium length cylindrical roots (K6); Imperator type with slender, short-conic-tipped roots (K7). Interestingly, accessions characterized by medium-length root of slightly pointed tip and broad shoulders, belonging to Danvers market type could not be assigned to any of the assumed clusters with a high level of confidence. 

The first three PCs explained only a small fraction of the total variation (PC1—8.44%; PC2—6.98%; PC3—5.25%). The Tracy–Widom statistics indicated that 49 PCs were significant at P = 0.05. Comparison of the inter- and intra-population diversity, where plants from the same cultivar were assigned to the same population, revealed that more than 64% of the total molecular variance was attributed to the ‘within population’ variance (Supplementary Figure S2). This was expected, as open pollinated carrot cultivars are genetically heterogenic, despite their satisfactory morphological uniformity. Thus, it was justified to use plants from the same open-pollinated cultivar as independent individuals for the association analysis.

### 2.3. GWAS

**Figure 2:**
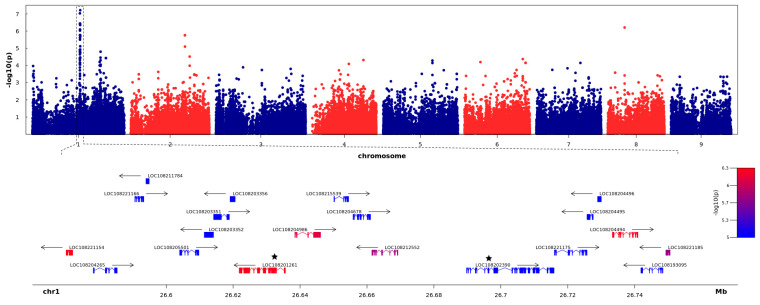
Manhattan Plot of association statistics and genes located within the chromosome 1 region associated with the shoulder diameter. The top panel shows the negative log10 *p*-values of SNPs, used to test associations based on the mixed linear model (MLM) for the carrot root shoulder diameter (*y*-axis), plotted against SNP positions on the nine carrot chromosomes (*x*-axis). The bottom panel shows genes annotated in the region associated with the shoulder diameter. Genes are colored based on the -log10 *p*-value of the most strongly associated SNP positioned within those genes. The two candidate genes are labeled with asterisks.

As no clear population structure was observed, we performed GWAS using a set of different parameters, i.e., PC1 to PC10, PC49, and the Bayesian model-based structure Q matrix (as inferred by STRUCTURE), in the MLM model. Despite the fact that different population structure estimates were applied, all GWAS results showed similar patterns of −log10(*p*-value) peaks on Manhattan plots. However, significance levels of the association differed, with the lowest *p*-values observed for PC5, so we performed the final analysis using the first five PCs (PC5). As a significance cut-off level, we used −log10(*p*-value) of Bonferroni-corrected *p*-value 0.05 equal to 6.18. Of the 81,748 SNPs, GWAS revealed seven SNPs that are significantly associated with the root shoulder diameter, a parameter reflecting carrot root width. Of those, six SNPs explaining approximately 10% of the total phenotypic variance clustered within one region of Chromosome 1 (Figure 2, Supplementary Table S2) while the remaining SNP was localized in the exon of LOC108198121 (encoding protein BIG GRAIN 1-like A) on chromosome 8 (Chr8_9416357). In the latter case, the associated region did not extend beyond the gene mentioned above, and it was not differentially expressed between cultivated and wild carrots nor during the carrot root development. Therefore, we focused on the region on chromosome 1 encompassing the six associated SNPs, which was located between positions 26,570,090 and 26,750,786, according to the DH1 carrot reference assembly [20], and comprised 20 genes spanning ca. 180 kb (Supplementary Table S3). Most of the 88 SNPs (67%) mapping to that region were localized within exons, while 13% were within introns, and 18% were intergenic (Supplementary Table S3). Of the six SNPs significantly associated with the carrot shoulder diameter, five were localized in exons and one was positioned 178 nt upstream of LOC108221185. Three of the five exonic SNPs were in exon 11 of LOC108201261, while the other two were in exon 1 of LOC108221154 and exon 2 of LOC108204494 (Figure 2, Supplementary Table S4). 

For the other root parameters, we did not identify SNPs meeting the Bonferroni-corrected significance threshold value. There were, however, regions with SNPs of −log10(*p*-value) above 4.5, that might be worth mentioning. Six SNPs revealed five regions on five chromosomes that might be associated with the collar diameter and five SNPs pointed at four regions located on four chromosomes, possibly determining root length (Supplementary Table S2, Supplementary Figure S3).

### 2.4. Identification and Verification of the Candidate Gene

Using previously reported transcriptomic data [14], we analyzed expression patterns of the 20 genes localized within the genomic region defined by GWAS, at different stages of development of cultivated and wild carrot roots. We searched for genes showing temporal expression changes in the cultivated carrots, but no changes in expression at corresponding time-points in wild carrots, as well as those showing differential expression between cultivated and wild carrots. Among the 20 genes localized in the genomic region of primary interest on Chromosome 1 (Figure 2), 11 were differentially expressed between one or more developmental stages (Supplementary Figure S4). We found one gene (LOC108203356) less expressed in cultivated carrots as compared to wild carrots at T1 and two genes (LOC108201261, LOC108202390) showing significantly increasing expression from T1 to T3 only in cultivated carrots. The remaining DEGs were up-regulated (LOC108204265) or down-regulated (LOC108204494, LOC108211784, LOC108215539) in the course of root development both in cultivated and wild carrots. Based on the expression patterns and the presumed function of the three DEGs differentiating wild and cultivated carrots, LOC108201261 (*DcDCAF1*), encoding DDB1-CUL4-associated factor 1, and LOC108202390 (*DcBTAF1*), encoding TATA-binding protein-associated factor 1, were the most likely candidate genes to be involved in root developmental processes. Both genes showed elevated expression in developing storage roots of cultivated carrots, as compared to wild carrots. In *Arabidopsis* homologs of these candidate genes are single-copy genes, while in carrot *DcBTAF1* is a single-copy gene, whereas in the case of *DcDCAF1* we have identified a paralog, i.e., LOC108203173 (*DcDCAF1l*), annotated as *DDB1-CUL4-associated factor homolog 1-like*, localized on chromosome 9 (position 31,511,550-31,522,821). Protein sequence identity between both carrot *DcDCAF1* paralogs was 74.5%. Even though the protein sequence identity between both carrot *DCAF1* paralogs was relatively high, we observed no association between *DcDCAF1l* on chromosome 9 with carrot root growth and no differential expression of *DcDCAF1l* was detected in root transcriptomes [14]. 

### 2.5. Genotyping and Expression of Candidate Genes in the Developing Storage Roots

**Figure 3:**
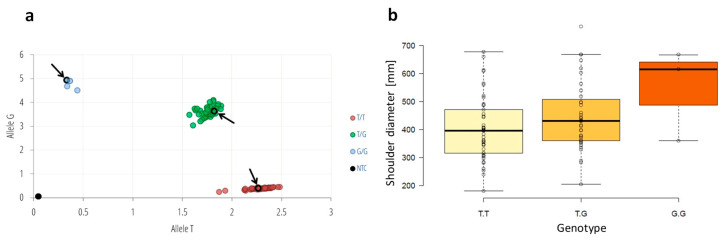
Genotyping and phenotyping of the three carrot half-sib populations; (**a**) allele discrimination plot of the SD_SNP_ (CHR1_26632616) genotyping assay of T/G polymorphism conducted on 90 plants from three populations produced through mass pollination; (**b**) boxplot showing differences in shoulder diameter in plants carrying different SD_SNP_ variants. The allelic discrimination plot was created as a graph of the normalized reporter signal for allele T probe (x-axis) plotted against the normalized reporter signal from the allele G probe (y-axis). Arrows and circled dots denote plants with known genotypes used as controls.

To confirm the functional association of the chromosome 1 region comprising *DCAF1* and *BTAF1* with carrot root width we developed an SD_SNP_ (shoulder diameter SNP at position CHR1_26632616, in intron 3 of *DcDCAF1*) genotyping assay based on the TaqMan technology and validated it on 90 plants from three half-sib carrot populations. In total, we identified 48 T/T homozygotes, 3 G/G homozygotes and 39 T/G heterozygotes (Figure 3). G/G genotypes were observed only in the population TCRS60. Distribution of T/T and T/G genotypes was similar for both TCRS56 and TCRS60 populations (ca. 36–43% of T/T and 53–57% of T/G). T/T genotypes were prevalent in TCRS72 population (80% of T/T and 20% of T/G). Tukey’s honestly significant difference (HSD) test showed significant difference between shoulder diameter measured for G_ and TT genotypes (*p* = 4 × 10^−6^).

**Figure 4:**
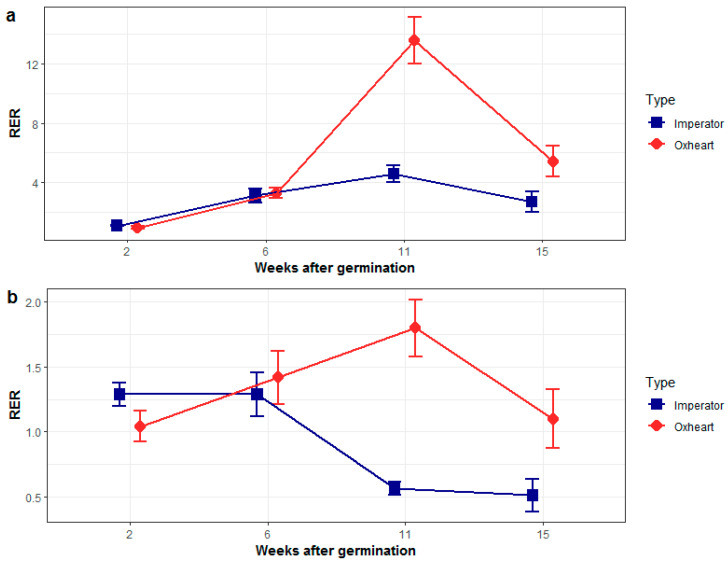
Relative expression of *DcDCAF1* (**a**) and *DcBTAF1* (**b**) at different developmental stages in two carrot varietal groups; an Imperator type (blue line) and an Oxheart type (red line).

We investigated the expression patterns of *DcDCAF1* and *DcBTAF1* throughout carrot storage root development in cultivars of Oxheart and Imperator varietal groups, characterized by contrasting root phenotypes and carrying different variants of the shoulder diameter-associated region on chromosome 1, as revealed by the SD_SNP_ genotyping assay (Supplementary Table S5). Contrasting SD_SNP_ homozygotes were used to determine *DcDCAF1* and *DcBTAF1* expression profiles in developing roots. For *DcDCAF1*, we observed an increase in expression, with the maximum reached at the secondary root growth phase, at which the root grows thicker (Figure 4a). Interestingly, in Imperator carrots, characterized by relatively long and thin storage roots, the increase was much less pronounced than that observed in Oxheart carrots producing shorter and much thicker roots. Subsequently, *DcDCAF1* was down-regulated in mature roots of both varietal types. 

The expression pattern of *DcBTAF1* in roots of Oxheart and Imperator carrots also differed markedly, even though *DcBTAF1* transcripts were generally less abundant than those of *DcDCAF1*. In Imperator carrots, *DcBTAF1* expression was gradually decreasing from T2 to T4, while in Oxheart carrots it was steadily increasing from T1 to T3, throughout the whole development of the storage root, while it eventually decreased in T4, after the mature storage root was formed (Figure 4b).

## 3. Discussion

Genetic mechanisms determining the observed diversity of shapes and sizes of carrot storage roots are poorly recognized. In an earlier genetic analysis of root shape, Turner et al. [8] identified several genetic determinants of the carrot root shape on Chromosome 1, including QTL for root length, root tapering, and tip fill. However, no QTLs related to root width were noted and the three Chromosome 1 QTL they reported were more proximal to the centromere than the 180 kb region discovered in this study. Parental lines in that earlier study all had relatively narrow shoulder diameters compared to many entries in the current study, and low repeatability in measuring several root trait parameters was reported. These differences may have limited their accurate detection of root thickening genes. It emphasizes the importance of the fact that, in our GWAS, we used a collection of accessions showing much larger diversity with respect to the storage root morphology.

The complexity of responses to environmental conditions makes the analysis of genetic factors governing the root morphology very challenging. In addition, as carrot suffers from inbreeding depression, there are very few homozygous lines available for research. Unlike many other crops, association studies in carrot must therefore rely on collections of accessions comprising mostly open-pollinated cultivars with high levels of intra-population variability. Here, we have shown that, indeed, most variability was attributed to differences within accessions and no apparent genetic diversity structure was revealed, which prompted us to use phenotyping and genotyping data of individual plants for GWAS. It has inevitably limited the power to discriminate genomic regions governing storage root morphology. We conclude that the carrot root shape is controlled by QTLs of minor effects, mostly falling below the significance threshold level, because of the experimental error when phenotyping individual plants. Beside the QTL for shoulder diameter on chromosome 1, discussed below, we observed a number of signals not reaching the significance threshold, associated with other parameters and possibly pointing at genomic regions possibly involved in root shape determination. 

Nevertheless, we were able to reveal a 180 kb-long genomic region on chromosome 1, comprising 20 genes including putative candidates affecting carrot secondary root growth. To identify candidate genes within the region revealed by GWAS, we analyzed the expression patterns of genes annotated in that region. Three genes showed expression changes related to phases of the storage root development. Based on their biological function, *DcDCAF1* and *DcBTAF1* were considered the most relevant. The remaining DEG, LOC108203356, encoded RuBisCO accumulation factor 1 (RAF1). RAF1 is a RuBisCO assembly chaperone protein required for the assembly and stability of RuBisCo [21]. *RAF1* transcripts were the most abundant at the seedling stage, where the total RNA from the whole plant was used for RNAseq [14]. As *RAF1* in *A. thaliana* is mostly expressed in leaves [22], its differential expression in carrot seedlings was likely not associated with the root developmental process and the gene was not considered a candidate. 

Analysis of a putative function of two other genes revealed their involvement in hormone-dependent regulatory processes that might be important for carrot storage root development. Starting from growth and development of the root apical meristem, which contributes to root elongation, hormones are key factors regulating root development. Auxins regulate a stem cell niche (SNC) activity and meristem growth, cytokinins are responsible for the induction of cell differentiation at the transition zone (TZ), and gibberellins can selectively repress cytokinin-responsive transcription factors [23]. The radial root growth depending on the secondary meristem called vascular cambium, which differentiates into xylem and phloem, is regulated by the major plant hormones [24]. A comprehensive analysis of the dynamics of hormones and gene expression changes during carrot root development is still missing. However, recent reports on differential expression of genes related to plant hormone metabolism and signaling during carrot root development point at the stage-dependent regulation of that process by different hormones [25,26]. Cytokinin concentration during carrot root development peaked at the stage of elongation and rapidly decreased at the beginning of secondary root growth, suggesting that different hormone-related genes were involved in the formation of carrot storage roots [27]. A comparison of transcriptomic changes during carrot root development between wild and cultivated plants indicated that transcription factors and genes encoding proteins involved in post-translational modifications (signal transduction and ubiquitination, including *DcDCAF1*) were up-regulated during storage root formation [14], and differential expression of homeobox genes during carrot root growth and development suggested that WOX and KNOX subfamilies are likely involved in carrot root development [28].

DCAF1 belongs to the class of WD40 proteins called DCAF functioning as substrate receptors mediating ubiquitination of specific proteins [29]. Both carrot DCAF1 proteins carry two WDxR motifs essential for their interaction with DDB1, suggesting that both might be functionally involved in the recognition of specific proteins. In *Arabidopsis*, *DCAF1* is expressed in most tissues and developmental stages and its knock-out mutations were reported to cause embryogenic lethality at the late globular stage, suggesting its participation in a range of biological processes [30]. It was further shown that DCAF1 protein functions as a negative regulator of ABA signaling [31]. The ABA signaling pathway is a complex system participating in the regulation of plant growth and development, including the development of the root system. The precise control of ABA signaling is achieved by post-translational modifications of ABA-dependent transcription factors [32,33]. DCAF1 functions as a substrate receptor in the CUL4-DDB1 machinery ubiquitinating ABI5 (abscisic acid-insensitive 5) when it is no longer needed or when its levels should be reduced [31]. ABI5 is a bZIP transcription factor functioning in the core ABA signaling and affects seed germination, seedling development, growth of lateral roots and other developmental processes [34]. Exogenous ABA negatively affects lateral root development [35]. In carrot, loss of the lateral root branching is considered as one of the domestication syndromes [9]. Moreover, in the sweet potato the levels of IAA and ABA were significantly increased in storage roots at the initial stage compared with the fibrous roots, and the levels declined gradually to reach the lowest point in mature roots. It was suggested that ABA was essential for tuber formation, and that it might play roles in storage root bulking by activating cell division [36]. We hypothesize that *DcDCAF1* in carrot might be reprogrammed to determine the secondary growth of the storage root by interaction with ABA responsive genes through ubiquitination of ABI5. 

The second candidate gene, *DcBTAF1*, differentially expressed throughout development of the storage root in the cultivated carrot, encodes BTAF1, a member of SWI2/SNF2-family ATPases, that is highly conserved among eukaryotes, usually encoded by a single-copy gene [37]. As with other proteins of that family, BTAF1 binds to TBP (TATA binding protein) in the presence or absence of DNA with the N-terminal HEAT/ARM repeats and contacts DNA upstream of TATA with its ATPase domain [37]. It was shown that BTAF1 changes the TATA-box specificity of TBP, and DNA-binding mutants of TBP can be rescued for the interaction with TATA box or TATA-less DNA by the addition of BTAF1 [38]. BTAF1 is involved in developmental processes in plants and belongs to phytohormone-responsive organogenesis-related genes involved in apical meristem development, particularly in the organization and maintenance of shoot apical meristem and maintenance of the root apical meristem. *Arabidopsis* rgd3 mutants of this gene showed impairment in root development at high temperatures if the mutation was localized in the middle of protein, between conserved domains, while seedlings with the mutation within HEAT repeats exhibited such phenotype constitutively [39]. Expression of that gene can be both up- and down-regulated by cytokinins during de novo shoot organogenesis in *Arabidopsis*, depending on the developmental stage [40]. Differential expression of the carrot *DcBTAF1* among thick- and thin-rooted cultivars and cultivated and wild carrots may suggest its involvement in the dose-related regulation of expression of downstream genes involved in storage root formation.

## 4. Materials and Methods 

A set of 103 open-pollinated carrot cultivars from the collection of genetic resources at Warwick Crop Centre, University of Warwick, Wellesbourne, UK, representing different root shape types (Supplementary Table S6), was kindly provided by Dr. C. Allender. They were grown in the field in PlantiCo Gołębiew, Poland, in 2016, under standard agricultural practice, optimally irrigated, fertilized and protected from pathogens. One to five plants from each cultivar, 327 plants in total, were phenotyped for root shape parameters and genotyped.

### 4.1. Phenotyping

Four parameters, i.e., root length (L), collar diameter (C) measured at the attachment of the leaf rosette, shoulder diameter (S) defined as the widest part of the root, and tip diameter (T) measured at ca. 15 mm from the bottom end of the storage root, were measured (Supplementary Figure S5). Based on these measurements, outlier plants, characterized by trait values outside 1.5× interquartile range above the upper quartile or below the lower quartile, were identified based on the Tukey box plot analysis in R v.3.6.1 [41], and removed from the datasets prior to the analysis. Pearson correlation coefficients were calculated in R v.3.6.1 [41], pairwise for all traits. 

### 4.2. Genotyping

DNA was extracted from young leaves using a modified CTAB protocol [42]. Plants were genotyped using genotyping-by-sequencing (GBS) at the University of Wisconsin-Madison Biotech Center, USA, essentially as described by Ellison et al. [11]. SNPs were called using the GBS pipeline in Tassel v.5.2.31 [43,44] and using the carrot DH1 genome (GenBank assembly accession number: GCA_001625215.1; [20]) as a reference. Two datasets, D1 and D2, were prepared by filtering polymorphisms in VCFtools v.0.1.14 [45]. The dataset D1, used to determine the population structure and calculate the kinship matrix, comprised high-quality SNPs (parameters: —max-alleles 2—max-missing-count 85—maf 0.4—minDP 8—thin100000). The dataset D2, used for GWAS, comprised SNPs filtered with the following parameters: —max-missing 0.5—max-alleles 2—maf 0.1—minDP 8. Subsequently, as proposed by Ellison et al. [11], sites with allele frequencies higher than 0.7 or lower than 0.3 were encoded as missing data and imputed using Beagle v.4.1 [46]. Following imputation, the dataset D2 was additionally filtered (—maf 0.1) and used for downstream analyses.

### 4.3. Population Structure

We used 1808 high-quality SNPs (D1) to detect and adjust for relatedness and population structure. Relatedness was corrected using Centered_IBS K-matrix calculated in Tassel v.5.2.31 [43]. To adjust data for population structure, we used the Bayesian model based on STRUCTURE v. 2.3.4 [47] and PCA-based matrix calculated in Tassel v.5.2.31. Using STRUCTURE v.2.3.4, we conduced five independent iterations of clustering with an admixture and independent allele frequencies model. We set the number of assumed populations (K values) from 1 to 11 and the length of burn-in to 100,000 followed by 500,000 Monte Carlo iterations. The most likely number of clusters (K) was estimated as described by Stelmach et al. [48]. To define the number of principal components needed to control population stratification, we performed Tracy–Widom statistics on PCA eigenvalues using ‘LEA‘ R package v.2.6.0 [49]. As the result indicated that 49 principle components were significant at the level of *p* < 0.05, we used PCA matrices comprising from two to ten, and 49 PCs to test for associations.

### 4.4. Association Analysis

GWAS was performed for 307 plants using 75,525 SNPs from the dataset D2 and four parameters describing the root shape. Associations were tested using mixed linear model (MLM) in Tassel v.5.2.31 [42]. The association significance threshold cut-off was defined based on Bonfferoni-corrected *p*-value = 0.05. GWAS results and the region associated with the root shoulder diameter were plotted using ‘Sushi’ R package v.1.18.0 [50]. 

### 4.5. Identification and Validation of Candidate Genes

To define the genomic region associated with shoulder diameter, we determined the positions of SNPs that were significantly associated with the trait and extended it to the positions of the two nearest non-significant SNPs at both sides. Next, we analyzed the expression patterns of all genes from the defined region throughout the root development period using previously reported transcriptomic data [14]. The candidate genes were defined based on their differential expression in the roots and the presumed biological function pointing at their possible involvement in the carrot root development. We used the sequences of a DCAF1 (XP_017225050) and BTAF1 (XP_017226283) proteins as a query for a tblastn search of NCBI ‘RefSeq Representative Genomes’ database, to identify homologous genes in carrot and *Arabidopsis thaliana*. 

### 4.6. Experimental Validation

To validate the association of the candidate genes with the shoulder diameter, we used populations obtained from mas-pollination of three maternal plants. Plants from two Paris Market type (RS56 and RS60) and one Imperator type (RS72) cultivars were used as mothers and mass-pollinated in the isolation cage with a mixture of pollen from ca. 60 plants representing cultivars of different types. In total, 30 plants of each progeny were phenotyped and genotyped (populations TCRS56, TCRS60, TCRS72).

#### 4.6.1. SNP Genotyping

SD_SNP_ genotyping (using SNP at position CHR1_26632616) was performed using Custom TaqMan^®^ Genotyping Assay (Applied Biosystems™; Thermo Fisher Scientific, Waltham, MA, USA). SD_SNP_ probes and primers flanking the analyzed locus were designed on commission (Supplementary Table S7). Amplification was carried out in 12 µL volume containing 2.8 ng of genomic DNA, 5 µL of TaqPath™ ProAmp™ Master Mix (Applied Biosystems™; Thermo Fisher Scientific, Waltham, MA, USA) and 0.5 µL of Custom TaqMan^®^ SNP Genotyping Assay mix (20-fold mix). Amplification protocol started with 95 °C (300 s) followed by 40 cycles of 95 °C (15 s) and 60 °C (60 s). Genotyping was performed using a 96-well QuantStudio 3 Real-Time PCR Thermalcycler (Applied Biosystems™; Thermo Fisher Scientific, Waltham, MA, USA), following the instructions of the supplier. 96-well plate contained 90 samples, 3 controls representing each allelic state: T/T, T/G, G/G, respectively, and 3 no-template controls. Genotypes were determined based on the dye-component fluorescent emission data depicted in the XY scatter-plot generated by Applied Biosystems™ (Thermo Fisher Scientific, Waltham, MA, USA) Analysis Software, Genotyping Analysis Module (version 3.9). Significance of differences between genotype–phenotype relationships was tested using Tukey’s HSD test in R v.3.6.1 [41]. 

#### 4.6.2. RT-qPCR

To analyze expression of the candidate genes, we grew plants from three Oxheart and three Imperator type cultivars, clearly differing in root morphology. Leaves and roots for genotyping and RT-qPCR, respectively, were collected from five plants representing each population at four time-points: T1—two weeks after germination (seedlings), T2—six weeks after germination (root elongation), T3—eleven weeks after germination (secondary root growth, thickening), T4—fifteen weeks after germination (mature storage roots). In total, we genotyped 117 plants (4–5 plants from each population collected at each time-point) using the SD_SNP_ assay, as described above. Next, at each time-point, following SD_SNP_ genotyping, we selected four homozygous plants representing Oxheart and Imperator cvs. and used them to evaluate expression of the candidate genes. The total RNA was extracted, purified, and reverse-transcribed according to Macko-Podgórni et al. [13]. Primers (Supplementary Table S8) used for analysis were designed based on the carrot reference genome [20] using Primer-BLAST [51] and verified with OligoAnalyzer (https://www.idtdna.com/pages/tools/oligoanalyzer; IDT; Coralville, IA, USA). To select the most reliable reference genes, we screened three chromosomal genes (actin7, LOC108202619; GADPH, LOC108223758; and EF-1-alpha, LOC108222822) and two plastid genes (rpl2, DCAR_032527; TIF-1, DCAR_032520). RT-qPCR was performed using the StepOnePlus Real-Time PCR (Thermo Fisher Scientific, Waltham, MA, USA). Primer efficiencies were calculated as described by Bowman et al. [52]. Based on the efficiency and stability of expression evaluated with BestKeeper v.1 [53], actin7 and TIF-1 were used as a reference. Relative expression ratios (RERs) were calculated using the ddCt method [54]. ANOVA followed by Tukey’s HSD post-hoc tests [55] were performed in R v.3.6.1 [41].

## 5. Conclusions

The carrot storage root morphology is controlled by many loci with minor effects. The most significant association with the shoulder diameter was found for a ca. 180 kb-long region on chromosome 1 comprising 20 genes of which three were differentially regulated throughout the root growth period. Based on their presumed function, two genes, LOC108201261, encoding DCAF1 (DDB1-CUL4 Associated Factor 1) and LOC108202390 encoding BTAF1 (TATA-binding protein-associated factor 1) were selected as the most likely candidates to be involved in the determination of root development. Two alleles of *DcDCAF1* were recognized by the presence of SNP revealed by the SD_SNP_ marker. While the developmental regulation of expression of *DcDCAF1* was essentially similar in all carrot cultivars, one of these alleles, present in Oxheart-type cultivars producing thick roots, showed much higher expression at the secondary root growth stage, as compared to the other allele, present in Imperator-type cultivars developing thinner and longer roots. *DcBTAF1* was also differentially regulated in cultivars producing long and thin roots as compared to those with thicker roots, being more highly expressed in the latter. The identification of genes potentially involved in the development of the carrot storage root provides a first insight into one of the possible mechanisms controlling the development of carrot storage roots and may facilitate carrot breeding with respect to root shape.

## Figures and Tables

**Table 1:** Descriptive statistics for analyzed phenotypic traits.

Trait	Mean [mm]	Range [mm]	SD	CV%
shoulder	41.73	19.42–73.76	11.06	26.51
length	213.86	60.00–365.00	57.99	27.11
collar	15.98	5.08–30.98	5.30	33.19
tip	21.64	5.25–46.04	8.17	37.79

## Supplementary Materials

Supplementary materials can be found at https://www.mdpi.com/1422-0067/21/12/4263/s1.

## Abbreviations

BTAF1	*TATA-binding protein-associated factor 1*
DCAF1	*DDB1-CUL4 Associated Factor 1*
GBS	genotyping-by-sequencing
GWAS	genome wide association studies
HSD	honestly significant difference
LD	linkage disequilibrium
QTL	Quantitative traits loci
SNP	Single nucleotide polymorphism

## References

[1] Banga O. (1957). The development of the original European carrot material. Euphytica.

[2] Thompson R. (1969). Some factors affecting carrot root shape and size. Euphytica.

[3] White J.M. (1992). Carrot yield when grown under three soil water concentrations. Hort Sci..

[4] Evers A.M., Tuuri H., Hägg M., Plaami S., Häkkinen U., Talvitie H. (1997). Soil forming and plant density effects on carrot yield and internal quality. Plant. Foods Hum. Nutr.

[5] Sakamoto M., Suzuki T. (2015). Elevated root-zone temperature modulates growth and quality of hydroponically grown carrots. Agric. Sci..

[6] Mbatha A.N., Ceronio G.M., Coetzer G.M. (2014). Response of carrot (*Daucus carota* L.) yield and quality to organic fertiliser. S. Afr. J. Plant. Soil.

[7] Jagosz B. (2012). Combining ability of carrot (*Daucus carota* L.) lines and heritability of yield and its quality components. Folia Hortic..

[8] Turner S.D., Ellison S., Senalik D.A., Simon P.W., Spalding E., Miller N. (2018). An automated image analysis pipeline enables genetic studies of shoot and root morphology in carrot (*Daucus carota* L.). Front. Plant. Sci.

[9] Ellison S., Simon P.W., Iorizzo M., Grzebelus D., Baranski R. (2019). Carrot Domestication. The Carrot Genome.

[10] Grzebelus D., Iorizzo M., Senalik D., Ellison S., Cavagnaro P., Macko-Podgorni A., Simon P.W. (2014). Diversity, genetic mapping, and signatures of domestication in the carrot (*Daucus carota* L.) genome, as revealed by Diversity Arrays Technology (DArT) markers. Mol. Breed..

[11] Ellison S.L., Luby C.H., Corak K.E., Coe K.M., Senalik D., Iorizzo M., Dawson J.C. (2018). Carotenoid Presence Is Associated with the *Or* Gene in Domesticated Carrot. Genetics.

[12] Macko-Podgórni A., Iorizzo M., Smółka K., Simon P.W., Grzebelus D. (2014). Conversion of a diversity arrays technology marker differentiating wild and cultivated carrots to a co-dominant cleaved amplified polymorphic site marker. Acta Biochim. Pol..

[13] Macko-Podgórni A., Machaj G., Stelmach K., Senalik D., Grzebelus E., Iorizzo M., Grzebelus D. (2017). Characterization of a genomic region under selection in cultivated carrot (*Daucus carota* subsp. *sativus*) reveals a candidate domestication gene. Front. Plant. Sci..

[14] Machaj G., Bostan H., Macko-Podgórni A., Iorizzo M., Grzebelus D. (2018). Comparative transcriptomics of root development in wild and cultivated carrots. Genes.

[15] Tian F., Bradbury P.J., Brown P.J., Hung H., Sun Q., Flint-Garcia S., Buckler E.S. (2011). Genome-wide association study of leaf architecture in the maize nested association mapping population. Nat. Genet..

[16] Beyer S., Daba S., Tyagi P., Bockelman H., Brown-Guedira G., Mohammadi M. (2019). Loci and candidate genes controlling root traits in wheat seedlings—A wheat root GWAS. Funct. Integr. Genom..

[17] Huang X., Yang S., Gong J., Zhao Y., Feng Q., Gong H., Chen N. (2015). Genomic analysis of hybrid rice varieties reveals numerous superior alleles that contribute to heterosis. Nat. Comm..

[18] Keilwagen J., Lehnert H., Berner T., Budahn H., Nothnagel T., Ulrich D., Dunemann F. (2017). The Terpene Synthase Gene Family of Carrot (*Daucus carota* L.): Identification of QTLs and Candidate Genes Associated with Terpenoid Volatile Compounds. Front. Plant. Sci..

[19] Jourdan M., Gagné S., Dubois-Laurent C., Maghraoui M., Huet S., Suel A., Geoffriau E. (2015). Carotenoid content and root color of cultivated carrot: A candidate-gene association study using an original broad unstructured population. PLoS ONE.

[20] Iorizzo M., Ellison S., Senalik D., Zeng P., Satapoomin P., Huang J., Yildiz M. (2016). A high-quality carrot genome assembly provides new insights into carotenoid accumulation and asterid genome evolution. Nat. Genet..

[21] Whitney S.M., Birch R., Kelso C., Beck J.L., Kapralov M.V. (2015). Improving recombinant Rubisco biogenesis, plant photosynthesis and growth by coexpressing its ancillary RAF1 chaperone. Proc. Natl. Acad. Sci. USA.

[22] Rhee S.Y., Beavis W., Berardini T.Z., Chen G., Dixon D., Doyle A., Miller N. (2003). The Arabidopsis Information Resource (TAIR): A model organism database providing a centralized, curated gateway to Arabidopsis biology, research materials and community. Nucleic Acids Res..

[23] Perilli S., Di Mambro R., Sabatini S. (2012). Growth and development of the root apical meristem. Curr. Opin. Plant. Biol..

[24] Elo A., Immanen J., Nieminen K., Helariutta Y., Davey J. (2009). Stem cell function during plant vascular development. Seminars in Cell & Developmental Biology.

[25] Wang G.L., Jia X.L., Xu Z.S., Wang F., Xiong A.S. (2015). Sequencing, assembly, annotation, and gene expression: Novel insights into the hormonal control of carrot root development revealed by a high-throughput transcriptome. Mol. Genet. Genom..

[26] Wang G., Huang W., Li M., Xu Z., Wang F., Xiong A. (2016). Expression profiles of genes involved in jasmonic acid biosynthesis and signaling during growth and development of carrot. Acta Biochim. Biophys. Sin..

[27] Wang G.L., Sun S., Xing G.M., Wu X.J., Wang F., Xiong A.S. (2015). Morphological characteristics, anatomical structure, and gene expression: Novel insights into cytokinin accumulation during carrot growth and development. PLoS ONE.

[28] Que F., Wang G.L., Li T., Wang Y.H., Xu Z.S., Xiong A.S. (2018). Genome-wide identification, expansion, and evolution analysis of homeobox genes and their expression profiles during root development in carrot. Funct. Integr. Genom..

[29] Zhang Y., Feng S., Chen F., Chen H., Wang J., McCall C., Deng X.W. (2008). Arabidopsis DDB1-CUL4 ASSOCIATED FACTOR1 forms a nuclear E3 ubiquitin ligase with DDB1 and CUL4 that is involved in multiple plant developmental processes. Plant. Cell.

[30] Zhang Y., Feng S., Terzaghi W., Deng X.W. (2008). A new family of plant E3 ubiquitin ligases. Plant. Signal. Behav..

[31] Seo K.I., Lee J.H., Nezames C.D., Zhong S., Song E., Byun M.O., Deng X.W. (2014). ABD1 is an Arabidopsis DCAF substrate receptor for CUL4-DDB1–based E3 ligases that acts as a negative regulator of abscisic acid signaling. Plant. Cell.

[32] Yu F., Wu Y., Xie Q. (2016). Ubiquitin–proteasome system in ABA signaling: From perception to action. Mol. Plant..

[33] Zhang J., Hafeez M.T., Di D., Wu L., Zhang L. (2019). Precise control of ABA signaling through post-translational protein modification. Plant. Growth Regul..

[34] Skubacz A., Daszkowska-Golec A., Szarejko I. (2016). The role and regulation of ABI5 (ABA-Insensitive 5) in plant development, abiotic stress responses and phytohormone crosstalk. Front. Plant. Sci..

[35] Khan M.A., Gemenet D.C., Villordon A. (2016). Root system architecture and abiotic stress tolerance: Current knowledge in root and tuber crops. Front. Plant. Sci..

[36] Dong T., Zhu M., Yu J., Han R., Tang C., Xu T., Li Z. (2019). RNA-Seq and iTRAQ reveal multiple pathways involved in storage root formation and development in sweet potato (*Ipomoea batatas* L.). BMC Plant. Biol..

[37] Koster M.J., Snel B., Timmers H.T.M. (2015). Genesis of chromatin and transcription dynamics in the origin of species. Cell.

[38] Klejman M.P., Zhao X., van Schaik F.M., Herr W., Timmers H.T.M. (2005). Mutational analysis of BTAF1–TBP interaction: BTAF1 can rescue DNA-binding defective TBP mutants. Nucleic Acids Res..

[39] Tamaki H., Konishi M., Daimon Y., Aida M., Tasaka M., Sugiyama M. (2009). Identification of novel meristem factors involved in shoot regeneration through the analysis of temperature-sensitive mutants of Arabidopsis. Plant. J..

[40] Ćosić T., Raspor M., Savić J., Cingel A., Matekalo D., Zdravković-Korać S., Ninković S. (2019). Expression profiles of organogenesis-related genes over the time course of one-step de novo shoot organogenesis from intact seedlings of kohlrabi. J. Plant. Physiol..

[41] R Core Team (2014). R: A Language and Environment for Statistical Computing.

[42] Briard M., Le Clerc M., Grzebelus D., Senalik D., Simon P.W. (2000). Modified protocols for rapid carrot genomic DNA extraction and AFLP analysis using silver staining or radioisotopes. Plant. Mol. Biol. Rep..

[43] Bradbury P.J., Zhang Z., Kroon D.E., Casstevens T.M., Ramdoss Y., Buckler E.S. (2007). TASSEL: Software for association mapping of complex traits in diverse samples. Bioinformatics.

[44] Glaubitz J.C., Casstevens T.M., Lu F., Harriman J., Elshire R.J., Sun Q., Buckler E.S. (2014). TASSEL-GBS: A High Capacity Genotyping by Sequencing Analysis Pipeline. PLoS ONE.

[45] Danecek P., Auton A., Abecasis G., Albers C.A., Banks E., DePristo M.A., McVean G. (2011). The variant call format and VCFtools. Bioinformatics.

[46] Browning S.R., Browning B.L. (2007). Rapid and accurate haplotype phasing and missing data inference for whole genome association studies by use of localized haplotype clustering. Am. J. Hum. Genet..

[47] Pritchard J.K., Stephens M., Rosenberg N.A., Donnelly P. (2000). Association mapping in structured populations. Am. J. Hum. Genet..

[48] Stelmach K., Macko-Podgórni A., Machaj G., Grzebelus D. (2017). Miniature inverted repeat transposable element insertions provide a source of intron length polymorphism markers in the carrot (*Daucus carota* L.). Front. Plant. Sci..

[49] Frichot E., François O. (2015). LEA: An R package for landscape and ecological association studies. Methods Ecol. Evol..

[50] Phanstiel D.H., Boyle A.P., Araya C.L., Snyder M.P., Sushi R. (2014). Flexible, quantitative and integrative genomic visualizations for publication-quality multi-panel figures. Bioinformatics.

[51] Ye J., Coulouris G., Zaretskaya I., Cutcutache I., Rozen S., Madden T.L. (2012). Primer-BLAST: A tool to design target-specific primers for polymerase chain reaction. BMC Bioinform..

[52] Bowman M.J., Willis D.K., Simon P.W. (2014). Transcript abundance of phytoene synthase 1 and phytoene synthase 2 is associated with natural variation of storage root carotenoid pigmentation in carrot. J. Am. Soc. Hortic Sci..

[53] Pfaffl M.W., Tichopad A., Prgomet C., Neuvians T.P. (2004). Determination of stable housekeeping genes, differentially regulated target genes and sample integrity: BestKeeper–Excel-based tool using pair-wise correlations. Biotechnol. Lett..

[54] Livak K.J., Schmittgen T.D. (2001). Analysis of relative gene expression data using real-time quantitative PCR and the 2− ΔΔCT method. Methods.

[55] Tukey J.W. (1949). Comparing individual means in the analysis of variance. Biometrics.

