# Supplementary material for: Mining for Candidate Genes Controlling Secondary Growth of the Carrot Storage Root
Source: Int J Mol Sci. 2020 Jun 15;21(12):4263. doi: 10.3390/ijms21124263 (PMC7352697; doi:10.3390/ijms21124263)
Supplement: Supplementary file 1 [file ijms-21-04263-s001.pdf]

Supplementary table 1. Descriptive statistics for analyzed phenotypic traits in carrot varietal groups

Type	Mean [mm]				Range [mm]				SD				CV%			
	Shoulder	Length	Collar	Tip	Shoulder	Length	Collar	Tip	Shoulder	Length	Collar	Tip	Shoulder	Length	Collar	Tip
CARROT-AMSTERDAM	32.25	197.79	11.34	21.33	20.29-58.57	124.00-335.00	5.08-30.69	37.77-11.23	8.38	55.49	5.33	6.06	25.98	28.06	47.00	28.40
CARROT-AUTUMN KING	40.21	241.03	17.42	20.41	24.50-59.87	155.00-318.00	7.65-30.88	30.43-6.58	8.00	51.68	6.02	7.31	19.89	21.44	34.58	35.79
CARROT-BABY NANTES	33.03	196.67	13.55	17.16	29.24-30.60	170.00-180.00	8.98-10.79	18.94-9.77	5.43	37.86	6.41	6.68	16.44	19.25	47.29	38.93
CARROT-BERLIC/IMPER	29.04	166.33	10.31	12.86	28.12-31.70	160.00-170.00	8.62-12.50	20.90-10.34	1.07	5.51	1.84	2.28	3.67	3.31	17.86	17.71
CARROT-BERLICUM	34.81	239.48	14.42	20.62	19.42-65.90	145.00-365.00	9.91-23.96	33.17-10.95	6.26	51.39	3.66	5.71	17.99	21.46	25.40	27.69
CARROT-CHANTENAY	53.51	201.21	17.76	29.08	33.56-73.76	125.00-320.00	8.74-26.45	39.00-20.54	10.22	41.65	4.82	5.14	19.11	20.70	27.16	17.68
CARROT-DANVERS	47.89	223.39	17.71	19.88	33.56-71.34	125.00-265.00	8.74-30.98	30.31-17.24	7.62	28.03	5.56	5.02	15.91	12.55	31.37	25.24
CARROT-EARLY SH HORN	54.03	117.95	16.97	31.98	28.95-73.06	60.00-230.00	10.44-27.00	46.04-8.83	10.61	44.23	5.25	7.76	19.64	37.50	30.95	24.26
CARROT-FLAKKEE	37.56	227.50	14.04	20.14	30.74-59.44	190.00-255.00	8.77-17.36	30.57-11.73	6.38	28.42	3.11	6.95	17.00	12.49	22.19	34.49
CARROT-GUERANDE	53.97	120.00	16.06	30.13	37.62-73.58	90.00-225.00	9.85-28.80	44.62-11.21	12.24	20.11	5.75	10.57	22.68	16.76	35.82	35.08
CARROT-IMPERATOR	37.77	263.64	16.88	17.13	26.19-53.99	175.00-300.00	10.16-25.64	27.79-9.91	7.44	28.58	4.28	4.03	19.69	10.84	25.34	23.51
CARROT-LA MERVEILLE	38.35	183.75	13.24	17.53	28.55-45.79	130.00-225.00	10.36-14.59	23.41-9.98	6.84	51.21	3.37	5.70	17.84	27.87	25.45	32.52
CARROT-LONG CHANT	42.91	187.50	15.88	25.48	40.98-43.76	185.00-360.00	12.39-18.85	35.05-19.20	2.31	2.89	2.94	6.75	5.38	1.54	18.53	26.50
CARROT-LONG ORANGE	42.83	301.67	11.94	12.77	41.61-57.56	245.00-300.00	9.95-15.31	33.57-12.14	1.12	57.52	2.93	0.61	2.61	19.07	24.55	4.78
CARROT-M DE PARIS	56.82	77.50	12.30	32.80	56.08-39.19	75.00-240.00	9.7-19.30	19.3-32.02	1.05	3.54	3.68	1.10	1.84	4.56	29.89	3.34
CARROT-NANTES	33.12	212.14	13.50	23.86	24.64-44.84	110.00-265.00	7.16-20.68	38.63-13.81	4.19	35.61	3.23	6.62	12.66	16.79	23.96	27.74
CARROT-ST VALERY	43.87	254.62	17.82	10.08	31.48-56.36	205.00-300.00	10.48-27.88	18.79-5.25	8.61	23.32	5.03	4.01	19.64	9.16	28.20	39.76
COW CARROT	39.00	218.75	16.45	17.82	25.37-43.86	190.00-300.00	14.01-22.83	19.02-15.12	9.09	28.39	2.84	1.82	23.31	12.98	17.24	10.21
FODDER CARROT	42.75	248.93	20.37	15.30	26.70-60.73	180.00-320.00	12-29.70	32.86-8.03	8.61	34.36	4.79	5.97	20.14	13.80	23.52	38.99

Supplementary table 2. Positions of SNPs possibly associated with carrot root shape, with Bonferroni-corrected -log_{10}(p values) more than 4.5 but below the significance threshold.

Trait	Chr.	Position	-Log10(p)	R2	Nearest gene	Position	Annotation
collar	2	32538336	5.10	0.07	LOC108208831	exon	serine/threonine-protein kinase prpf4B
collar	4	29896504	4.70	0.07	LOC108218649	exon	protein QUIRKY
collar	5	34784932	5.08	0.07	LOC108223891	intron	anthranilate synthase alpha subunit 2, chloroplastic-like
collar	6	21186693	5.09	0.07	LOC108226406	intron	recQ-mediated genome instability protein 1
collar	7	35485532	4.68	0.07	LOC108196402	exon	zinc phosphodiesterase ELAC protein 2
collar	7	35485534	4.68	0.07	LOC108196402	exon	zinc phosphodiesterase ELAC protein 2 (tRNAse Z4 Arabidopsis)
length	1	29218354	4.83	0.07	LOC108226074	exon	probable galacturonosyltransferase-like 4
length	4	14697122	4.59	0.06	LOC108216034	exon	pentatricopeptide repeat-containing protein At2g39620
length	4	14697123	4.59	0.06	LOC108216034	exon	pentatricopeptide repeat-containing protein At2g39620
length	5	8096126	5.46	0.07	LOC108221094	exon	alkane hydroxylase MAH1-like
length	7	2584261	4.95	0.07	LOC108194423	exon	probable ascorbate-specific transmembrane electron transporter 1
shoulder	1	38140082	4.80	0.07	LOC108205299	exon	uncharacterized
shoulder	2	30248225	5.76	0.09	LOC108208010	5′UTR	RING-H2 finger protein ATL67-like
shoulder	2	30248233	5.76	0.08	LOC108208010	5′UTR	RING-H2 finger protein ATL67-like
shoulder	2	30269447	5.10	0.07	LOC108208468	intron	beta-amyrin 28-oxidase-like
shoulder	2	32901690	4.52	0.06	LOC108206064	exon	uncharacterized PKHD-type hydroxylase At1g22950-like

Supplementary table 3. List of genes located in the genomic region of chromosome 1, associated with the carrot root shoulder diameter.

Chr	Start	End	Gene	Annotation	No. SNPs	-LOG10(p)
chr1	26570090	26572044	LOC108221154	flotillin-like protein 3	21 (1)*	7.032928619
chr1	26578200	26585350	LOC108204265	hypothetical protein	0	
chr1	26590613	26593301	LOC108221166	polyadenylate-binding protein 8-like	0	
chr1	26593943	26594918	LOC108211784	uncharacterized	0	
chr1	26603883	26609759	LOC108205501	AT-hook motif nuclear-localized protein 14-like (AHL14)	0	
chr1	26611372	26614144	LOC108203352	uncharacterized	0	
chr1	26614162	26618875	LOC108203351	uncharacterized (EIN4)	2	
chr1	26619087	26620611	LOC108203356	rubisco accumulation factor 1, chloroplastic-like	2	
chr1	26621804	26635632	LOC108201261	DDB1- and CUL4-associated factor homolog 1 isoform X1 (DCAF1)	18 (3)	6.446505488
chr1	26638342	26646140	LOC108204986	filament-like plant protein 3	14	
chr1	26647924	26654507	LOC108215539	LOC108215539	0	
chr1	26655833	26661068	LOC108204678	cyclin-H1-1	0	
chr1	26661461	26669245	LOC108212552	ribonuclease H2 subunit B	2	
chr1	26689735	26715905	LOC108202390	TATA-binding protein-associated factor BTAF1	2	
chr1	26716017	26725762	LOC108221175	queuine tRNA-ribosyltransferase subunit qtrtd1-like	1	
chr1	26725753	26727618	LOC108204495	uncharacterized protein At4g15970-like	0	
chr1	26728912	26730033	LOC108204496	uncharacterized protein	3	
chr1	26733341	26740981	LOC108204494	uncharacterized protein	4 (1)	6.354105977
chr1	26741856	26748449	LOC108193095	uncharacterized protein	0	
chr1	26749314	26750604	LOC108221185	PTI1-like tyrosine-protein kinase 1	5	

*in parentheses; number of SNPs significantly associated with the shoulder diameter

Supplementary table 4. List of SNPs identified in the region on chromosome 1, associated with carrot root width. Significant $-log_{10}$(p values) are highlighted in red.

Chr1	Pos	Marker	Region	Feature	R2	$-Log_{10}(p)$
chr1	26570180	S1_26570180	LOC108221154	exon 1	0.061276	4.427434
chr1	26570182	S1_26570182	LOC108221154	exon 1	0.061276	4.427434
chr1	26570216	S1_26570216	LOC108221154	exon 1	0.004524	0.317455
chr1	26570246	S1_26570246	LOC108221154	exon 1	0.067583	4.896009
chr1	26570253	S1_26570253	LOC108221154	exon 1	0.053945	3.882012
chr1	26570256	S1_26570256	LOC108221154	exon 1	0.064249	4.646578
chr1	26570291	S1_26570291	LOC108221154	exon 1	0.008449	0.593727
chr1	26570309	S1_26570309	LOC108221154	exon 1	0.09632	7.032929
chr1	26570327	S1_26570327	LOC108221154	exon 1	0.007149	0.502839
chr1	26570353	S1_26570353	LOC108221154	exon 1	0.007509	0.527403
chr1	26570437	S1_26570437	LOC108221154	exon 1	0.004244	0.296853
chr1	26570451	S1_26570451	LOC108221154	exon 1	0.004244	0.296853
chr1	26570480	S1_26570480	LOC108221154	exon 1	0.006085	0.42594
chr1	26570535	S1_26570535	LOC108221154	exon 1	9.24E-04	0.064725
chr1	26570576	S1_26570576	LOC108221154	exon 1	0.008835	0.620222
chr1	26571083	S1_26571083	LOC108221154	exon 2	0.051748	3.720182
chr1	26571221	S1_26571221	LOC108221154	exon 2	0.057558	4.148658
chr1	26571485	S1_26571485	LOC108221154	exon 2	0.045028	3.225748
chr1	26571581	S1_26571581	LOC108221154	exon 2	0.033311	2.371938
chr1	26571590	S1_26571590	LOC108221154	exon 2	0.001958	0.137226
chr1	26571662	S1_26571662	LOC108221154	exon 2	0.059932	4.320792
chr1	26587834	S1_26587834	intergenic	-	0.003934	0.275791
chr1	26587867	S1_26587867	intergenic	-	0.003934	0.275791
chr1	26587873	S1_26587873	intergenic	-	0.056387	4.072053
chr1	26587886	S1_26587886	intergenic	-	0.057609	4.164419
chr1	26587887	S1_26587887	intergenic	-	0.057609	4.164419
chr1	26587924	S1_26587924	intergenic	-	0.066382	4.82002
chr1	26587969	S1_26587969	intergenic	-	0.003846	0.269889
chr1	26588608	S1_26588608	intergenic	-	0.00196	0.137327
chr1	26588620	S1_26588620	intergenic	-	0.002241	0.156953
chr1	26588639	S1_26588639	intergenic	-	9.87E-04	0.069077
chr1	26588656	S1_26588656	intergenic	-	0.002241	0.156953
chr1	26615554	S1_26615554	LOC108203351	exon 2	0.028188	1.996618
chr1	26615563	S1_26615563	LOC108203351	exon 2	0.001776	0.12425
chr1	26619182	S1_26619182	LOC108203356	exon 1	0.029606	2.110899
chr1	26619192	S1_26619192	LOC108203356	exon 1	0.061066	4.413726
chr1	26623951	S1_26623951	LOC108201261	exon 14	0.020383	1.443466
chr1	26624055	S1_26624055	LOC108201261	exon 14	0.022718	1.612486
chr1	26624496	S1_26624496	LOC108201261	exon 14	0.047254	3.389724
chr1	26624639	S1_26624639	LOC108201261	exon 14	0.002792	0.195493
chr1	26624681	S1_26624681	LOC108201261	exon 14	0.010363	0.73026
chr1	26624741	S1_26624741	LOC108201261	exon 14	0.006961	0.489066
chr1	26624851	S1_26624851	LOC108201261	intron 13	0.067999	4.922345
chr1	26627331	S1_26627331	LOC108201261	exon 11	0.076906	5.599963

chr1	26627382	S1_26627382	LOC108201261	exon 11	0.087739	6.408401
chr1	26627718	S1_26627718	LOC108201261	exon 11	0.087428	6.42187
chr1	26627751	S1_26627751	LOC108201261	exon 11	0.087748	6.446505
chr1	26627825	S1_26627825	LOC108201261	intron 9	0.049063	3.522271
chr1	26631200	S1_26631200	LOC108201261	intron 5	0.013017	0.913688
chr1	26631388	S1_26631388	LOC108201261	intron 5	0.039991	2.866688
chr1	26631424	S1_26631424	LOC108201261	exon 5	0.002472	0.173377
chr1	26631433	S1_26631433	LOC108201261	exon 5	0.056158	4.05085
chr1	26632616	S1_26632616	LOC108201261	intron 3	0.082554	6.030532
chr1	26632669	S1_26632669	LOC108201261	exon 3	0.075301	5.503109
chr1	26644332	S1_26644332	LOC108204986	exon 4	0.014409	1.013986
chr1	26645396	S1_26645396	LOC108204986	exon 4	0.026718	1.889774
chr1	26645532	S1_26645532	LOC108204986	exon 4	0.011054	0.777911
chr1	26645566	S1_26645566	LOC108204986	intron 4	5.59E-04	0.039093
chr1	26645570	S1_26645570	LOC108204986	intron 4	0.01022	0.718531
chr1	26645626	S1_26645626	LOC108204986	intron 4	0.011466	0.806827
chr1	26645629	S1_26645629	LOC108204986	intron 4	0.025056	1.779909
chr1	26645692	S1_26645692	LOC108204986	exon 5	0.065633	4.755355
chr1	26645710	S1_26645710	LOC108204986	exon 5	0.083012	6.098185
chr1	26645883	S1_26645883	LOC108204986	exon 5	0.021039	1.48772
chr1	26645896	S1_26645896	LOC108204986	exon 5	0.021039	1.48772
chr1	26646015	S1_26646015	LOC108204986	exon 5	0.012195	0.859798
chr1	26646019	S1_26646019	LOC108204986	exon 5	0.007875	0.55257
chr1	26646020	S1_26646020	LOC108204986	exon 5	0.007875	0.55257
chr1	26665284	S1_26665284	LOC108212552	intron 4	0.078722	5.770171
chr1	26665311	S1_26665311	LOC108212552	intron 4	0.078722	5.770171
chr1	26706998	S1_26706998	LOC108202390	exon 17	0.008132	0.569882
chr1	26708507	S1_26708507	LOC108202390	exon 19	0.061585	4.450148
chr1	26725064	S1_26725064	LOC108221175	intron 11	0.030819	2.198535
chr1	26728043	S1_26728043	intergenic	-	0.00231	0.161506
chr1	26729113	S1_26729113	LOC108204496	exon 1	0.002827	0.197937
chr1	26729155	S1_26729155	LOC108204496	exon 1	0.002827	0.197937
chr1	26729156	S1_26729156	LOC108204496	exon 1	0.002827	0.197937
chr1	26735248	S1_26735248	LOC108204494	exon 2	0.004286	0.299886
chr1	26735304	S1_26735304	LOC108204494	exon 2	0.086269	6.354106
chr1	26735325	S1_26735325	LOC108204494	exon 2	0.067696	4.929651
chr1	26735330	S1_26735330	LOC108204494	exon 2	0.007669	0.537434
chr1	26750333	S1_26750333	LOC108221185	exon 1	0.078193	5.736792
chr1	26750366	S1_26750366	LOC108221185	exon 1	0.039189	2.824098
chr1	26750440	S1_26750440	LOC108221185	exon 1	0.002466	0.172507
chr1	26750476	S1_26750476	LOC108221185	exon 1	0.003906	0.273771
chr1	26750515	S1_26750515	LOC108221185	exon 1	0.022599	1.598317
chr1	26750782	S1_26750782	intergenic	-	0.097088	7.216423
chr1	26750786	S1_26750786	intergenic	-	0.009497	0.666883
chr1	26750787	S1_26750787	intergenic	-	0.077805	5.688706
chr1	26751703	S1_26751703	intergenic	-	0.01329	0.939389
chr1	26751731	S1_26751731	intergenic	-	0.061592	4.469183
chr1	26751826	S1_26751826	intergenic	-	0.009148	0.643728
chr1	26751848	S1_26751848	intergenic	-	0.010633	0.750599

chr1	26751853	S1_26751853	intergenic	-	0.010633	0.750599
chr1	26760527	S1_26760527	LOC108204771	3'UTR	0.009253	0.65127
chr1	26760666	S1_26760666	LOC108204771	exon 2	0.035334	2.529766
chr1	26760672	S1_26760672	LOC108204771	exon 2	0.002288	0.160274

Supplementary table 5. Results of SD_{SNP} genotyping of plants from the Imperator and Oxheart varietal groups.

Oxheart			Imperator		
Plant	ID	**Genotype**	Plant	ID	**Genotype**
RS66_1	T1-1	G/G	RS73_1	T1-1	T/T
RS66_2	T1-2	G/G	RS73_2	T1-2	T/T
RS66_3	T1-3	G/G	RS73_3	T1-3	T/T
RS66_4	T1-4	G/G	RS73_4	T1-4	T/T
RS66_5	T1-5	G/G	RS73_5	T1-5	T/T
RS66_6	T2-1	G/G	RS73_6	T2-1	T/T
RS66_7	T2-2	G/G	RS73_7	T2-2	T/T
RS66_8	T2-3	G/G	RS73_8	T2-3	T/T
RS66_9	T2-4	G/G	RS73_9	T2-4	T/T
RS66_10	T2-5	G/G	RS73_10	x	x
RS66_11	T3-1	G/G	RS73_11	T3-1	T/T
RS66_12	T3-2	G/G	RS73_12	T3-2	T/T
RS66_13	T3-3	G/G	RS73_13	T3-3	T/T
RS66_14	T3-4	G/G	RS73_14	T3-4	T/T
RS66_15	T3-5	G/G	RS73_15	x	x
RS66_16	T4-1	G/G	RS73_16	T4-1	T/T
RS66_17	T4-2	G/G	RS73_17	T4-2	T/T
RS66_18	T4-3	G/G	RS73_18	T4-3	T/T
RS66_19	T4-4	G/G	RS73_19	T4-4	T/T
RS66_20	T4-5	G/G	RS73_20	T4-5	T/T
RS68_1	T1-1	G/G	RS75_1	T1-1	T/T
RS68_2	T1-2	G/G	RS75_2	T1-2	T/T
RS68_3	T1-3	G/G	RS75_3	T1-3	T/T
RS68_4	T1-4	G/G	RS75_4	T1-4	T/T
RS68_5	T1-5	G/G	RS75_5	T1-5	T/T
RS68_6	T2-1	G/G	RS75_6	T2-1	T/T
RS68_7	T2-2	G/G	RS75_7	T2-2	T/T
RS68_8	T2-3	G/G	RS75_8	T2-3	T/T
RS68_9	T2-4	G/G	RS75_9	T2-4	T/T
RS68_10	T2-5	G/G	RS75_10	T2-5	T/T
RS68_11	T3-1	G/G	RS75_11	T3-1	T/T
RS68_12	T3-2	G/G	RS75_12	T3-2	T/T
RS68_13	T3-3	G/G	RS75_13	T3-3	T/T
RS68_14	T3-4	G/G	RS75_14	T3-4	T/T
RS68_15	T3-5	G/G	RS75_15	T3-5	T/T
RS68_16	T4-1	G/G	RS75_16	T4-1	T/T

RS68_17	T4-2	G/G	RS75_17	T4-2	T/T
RS68_18	T4-3	G/G	RS75_18	T4-3	T/T
RS68_19	T4-4	G/G	RS75_19	T4-4	T/T
RS68_20	T4-5	G/G	RS75_20	T4-5	T/T
RS69_1	T1-1	G/G	RS76_1	T1-1	T/G
RS69_2	T1-2	T/G	RS76_2	T1-2	T/G
RS69_3	T1-3	T/T	RS76_3	T1-3	T/G
RS69_4	T1-4	T/G	RS76_4	T1-4	T/G
RS69_5	T1-5	G/G	RS76_5	T1-5	T/G
RS69_6	T2-1	G/G	RS76_6	T2-1	G/G
RS69_7	T2-2	G/G	RS76_7	T2-2	T/G
RS69_8	T2-3	T/G	RS76_8	T2-3	T/G
RS69_9	T2-4	T/G	RS76_9	T2-4	T/T
RS69_10	T2-5	G/G	RS76_10	T2-5	T/G
RS69_11	T3-1	T/G	RS76_11	T3-1	G/G
RS69_12	T3-2	T/G	RS76_12	T3-2	T/G
RS69_13	T3-3	T/G	RS76_13	T3-3	G/G
RS69_14	T3-4	G/G	RS76_14	T3-4	T/G
RS69_15	X	x	RS76_15	T3-5	T/T
RS69_16	T4-1	T/G	RS76_16	T4-1	T/G
RS69_17	T4-2	T/G	RS76_17	T4-2	T/G
RS69_18	T4-3	T/T	RS76_18	T4-3	T/T
RS69_19	T4-4	G/G	RS76_19	T4-4	T/G
RS69_20	T4-5	G/G	RS76_20	T4-5	T/T

Supplementary table 6. Origin of carrot plant used for GWAS

ID	WGRU	Name	Type	No. of plants (GBS)	No. of plants (GWAS)
RS01	7123	FORAM	CARROT-AMSTERDAM	3	3
RS02	3950	CYLINDRA AMSTERDAMER	CARROT-AMSTERDAM	2	2
RS03	3942	AMSTERDAM GRACE	CARROT-AMSTERDAM	3	2
RS04	3981	AMSTERDAM 5564	CARROT-AMSTERDAM	3	3
RS06	5477	AMSTERDAM FORCING	CARROT-AMSTERDAM	4	4
RS07	10371	AMSTERDAMSKA	CARROT-AMSTERDAM	4	4
RS08	6030	PICKMO	CARROT-AMSTERDAM	4	4
RS09	3970	AMSTEL	CARROT-AMSTERDAM	3	3
RS10	11153	AMSTERDAMMER BAK NORMAAL	CARROT-AMSTERDAM	4	4
RS11	6511	KAROTAN	CARROT-AUTUMN KING	4	4
RS12	10327	FLAKKEE	CARROT-AUTUMN KING	4	3
RS13	3961	FLAKKO	CARROT-AUTUMN KING	4	4
RS14	6513	AUTUMN KING VITA LONGA	CARROT-AUTUMN KING	3	3
RS15	6483	REGOL	CARROT-AUTUMN KING	3	2
RS16	3938	CENTURY	CARROT-AUTUMN KING	3	3
RS17	6021	ROTHILD	CARROT-AUTUMN KING	3	3
RS18	6064	VALORIA	CARROT-AUTUMN KING	2	2
RS19	6026	BEACON	CARROT-AUTUMN KING	3	3
RS20	6031	REGULUS IMPERIAL	CARROT-AUTUMN KING	4	4
RS21	8118	SUKO	CARROT-BABY NANTES	3	3
RS22	6484	FEONIA	CARROT-BERLIC/IMPER	3	3
RS23	10122	CYRANO	CARROT-BERLICUM	4	4
RS24	3949	BANTA	CARROT-BERLICUM	3	3
RS25	3851	PERFECTA	CARROT-BERLICUM	2	2
RS26	12403	LANGE ROTE ST O HERZ 2/ZINO	CARROT-BERLICUM	3	3
RS27	6085	BERLIKUM PERFECTA	CARROT-BERLICUM	4	4
RS29	6045	CAMBERLEY	CARROT-BERLICUM	3	3
RS30	11157	BERLICUM NORMAAL	CARROT-BERLICUM	3	3
RS31	8106	RONA	CARROT-BERLICUM	3	3
RS32	7256	FEONIA NOBO	CARROT-BERLICUM	4	4
RS33	8860	CHANTENAY ROYAL	CARROT-CHANTENAY	3	3
RS34	8847	CHANTENAY RED CORED	CARROT-CHANTENAY	4	4
RS35	3882	ROYAL CHANTENAY	CARROT-CHANTENAY	3	3
RS37	5127	GOLD KING	CARROT-CHANTENAY	4	4
RS38	11257	COMET	CARROT-CHANTENAY	3	3
RS39	5090	CHANTENAY LONG TYPE	CARROT-CHANTENAY	3	3
RS40	4670	MACBETH	CARROT-CHANTENAY	3	2
RS41	5589	CHANTENAY REX RS	CARROT-CHANTENAY	3	3
RS42	7255	CRIOLLA	CARROT-CHANTENAY	4	4
RS43	6487	DANVERS 126	CARROT-DANVERS	3	3
RS44	5595	DANVERS DANRO RS	CARROT-DANVERS	3	3
RS45	5128	DANVERS RED CORED	CARROT-DANVERS	4	4
RS46	6118	DANVERS HALF LONG E W K'S SCARLET INTERMEDIATE	CARROT-DANVERS	2	1
RS48	8097	DANVERS HALF LONG SCARLET INTERMEDIATE	CARROT-DANVERS	4	4
RS49	11144	DANVERS	CARROT-DANVERS	3	3
RS50	8098	DANVERS PRIDE	CARROT-DANVERS	3	3
RS51	8109	DANVERS HALF LONG	CARROT-DANVERS	4	4
RS52	10110	DANVERS HALF LONG DANVERS 126	CARROT-DANVERS	3	3
RS53	7131	DAVANTURE	CARROT-EARLY SH HORN	4	3
RS54	10414	EARLY SCARLET WONDER	CARROT-EARLY SH HORN	1	1
RS55	9308	DUWICKER	CARROT-EARLY SH HORN	3	2
RS56	5596	PARIS MARKET	CARROT-EARLY SH HORN	4	4
RS57	3966	PARIS FORCING	CARROT-EARLY SH HORN	3	1
RS58	6490	EARLY SCARLET HORN	CARROT-EARLY SH HORN	4	4
RS60	9294	PARIJSE MARKT	CARROT-EARLY SH HORN	4	4
RS61	9311	EARLY SCARLET HORN	CARROT-EARLY SH HORN	3	3
RS63	7259	COLORA	CARROT-FLAKKEE	2	2
RS64	7258	ROOD HILD	CARROT-FLAKKEE	4	4
RS65	6491	GUERANDE	CARROT-GUERANDE	3	1
RS66	13891	OXHEART	CARROT-GUERANDE	3	3
RS67	3983	FLATTIE (OXHEART)	CARROT-GUERANDE	4	3
RS68	9312	GUERANDE OXHEART	CARROT-GUERANDE	3	2
RS69	3906	OXHEART	CARROT-GUERANDE	3	3
RS70	5814	GOLD PAK 28	CARROT-IMPERATOR	4	4
RS71	3885	GOLD PAK	CARROT-IMPERATOR	4	4
RS72	3907	IMPERATOR 408	CARROT-IMPERATOR	3	3
RS73	11145	IMPERATOR	CARROT-IMPERATOR	4	4

RS74	3891	IMPERATOR 407	CARROT-IMPERATOR	1	1
RS75	3917	LONG IMPERATOR 58	CARROT-IMPERATOR	3	3
RS76	3892	IMPERATOR 58	CARROT-IMPERATOR	3	3
RS77	6769	ROUGE LA MERVEILLE	CARROT-LA MERVEILLE	4	4
RS78	3843	NEWMARKET	CARROT-LONG CHANT	4	4
RS79	6102	LONG RED SURREY	CARROT-LONG ORANGE	3	3
RS80	7121	KARDINAL MARCHE DE PARIS	CARROT-M DE PARIS	3	2
RS81	8874	EARLY NANTES	CARROT-NANTES	4	4
RS82	7125	DELTA A CUORE ROSSO	CARROT-NANTES	3	3
RS83	8895	TIP TOP	CARROT-NANTES	4	4
RS84	3956	TANTAL	CARROT-NANTES	3	3
RS85	3945	NANTES DUKE	CARROT-NANTES	4	4
RS86	5994	JUWAROT	CARROT-NANTES	4	4
RS87	6090	TOUCHON	CARROT-NANTES	4	4
RS88	7253	HILMAR	CARROT-NANTES	2	2
RS89	9328	SYTAN	CARROT-NANTES	4	4
RS90	6761	NANTSKAJA	CARROT-NANTES	3	3
RS91	3982	RED ELEPHANT	CARROT-ST VALERY	2	2
RS92	6514	ST VALERY	CARROT-ST VALERY	3	3
RS94	6788	ST VALERY	CARROT-ST VALERY	2	2
RS95	6004	KIELER ROTE (BAUERS)	CARROT-ST VALERY	2	2
RS96	6163	NEW RED INTERMEDIATE	CARROT-ST VALERY	4	4
RS97	13161	KOEWORTELEN	COW CARROT	4	4
RS98	6330	EGMONT GOLD	FODDER CARROT	3	3
RS99	10532	FLAKEE SAMO	FODDER CARROT	3	3
RS100	11290	FLAVIUS	FODDER CARROT	4	3
RS101	3924	LOBBERICHER	FODDER CARROT	3	3
RS102	10531	LANGE GELE KOE	FODDER CARROT	1	0
RS103	6488	LONG WHITE GREEN TOP	FODDER CARROT	2	1
RS104	3921	GELBE RHEINISCHE	FODDER CARROT	4	3
RS105	3918	BLANCHE A COLLET VERT TRES HORS TERRE	FODDER CARROT	1	0
RS106	11289	LOPEE	FODDER CARROT	4	3
RS107	9410	JAUNE OBTUSE DU DOUBS	FODDER CARROT	3	3
RS108	10530	LANGE GELE STOMPER	FODDER CARROT	3	3
RS109	8112	WHITE BELGIAN	FODDER CARROT	2	2
RS110	6787	SWHN-BIALA ZIELONOGLOWA	FODDER CARROT	1	1

Supplementary table 7. Sequences of primers and probes used for SD_{SNP} genotyping

SNP position	Primer	Primer sequence	fluorophore	Probe sequence	Allele
Chr1:26632616	forward	AGTTAGTGTTGCGCAGCTTATTAAA	VIC®	TTCGATTGCTACTTTGC	T
	reverse	CTGTTCATTGACGTGGATGGTATG	FAM™	TCGATTGCTCCTTTGC	G

Supplementary table 8. Sequences of primers used for expression analysis of *DcDCAF1* and *DcBTAF1*.

Name	Sequence	NCBI code	Phytozome code	Efficiency	R2	Product length
Actin7-q-F	GGTATTGTGTTGGACTCTGGTGAT	LOC108202619	DCAR_030967	95.203	1	96
Actin7-q-R	AGCAAGGTCAAGACGGAGTATG					
GADPH-q-F	GGGAGGTGCAAAGAAGGTTATCA	LOC108223758	DCAR_016414	94.893	0.999	79
GADPH-q-R	TTCCTTTTCATTGACACCAACAACA					
EF1A-q-F	GGCGATGCTGGATTCGTTAAG	LOC108222822	DCAR_019133	97.149	1	73
EF1A-q-R	GTGGAGGGTAGGACATGAAGG					
rpl2-q-F	CCTACCTTTGACCGATATGCCC	-	DCAR_032527	92.972	1	107
rpl2-q-R	TCAGTTTCGCTACAGCACCC					
TIF1-q-F	AAAACTACGTCGGATCTTTCCTGA	-	DCAR_032527	97.553	0.995	86
TIF1-q-R	CCCTTCCCAACGGTATGTTCC					
DCAF1-q-F	GTGGTGGGTTCCATCCAGC	LOC108201261	DCAR_002179	99.010	0.999	97
DCAF1-q-R	TCTAGGGAAGGCACACTACGA					
BTAF1-q-F	GACAACTCACACATAGAGGAT	LOC108202390	DCAR_002184	94.149	1	108
BTAF1-q-R	GTTAAAACGCTTCAAAAATGCT					

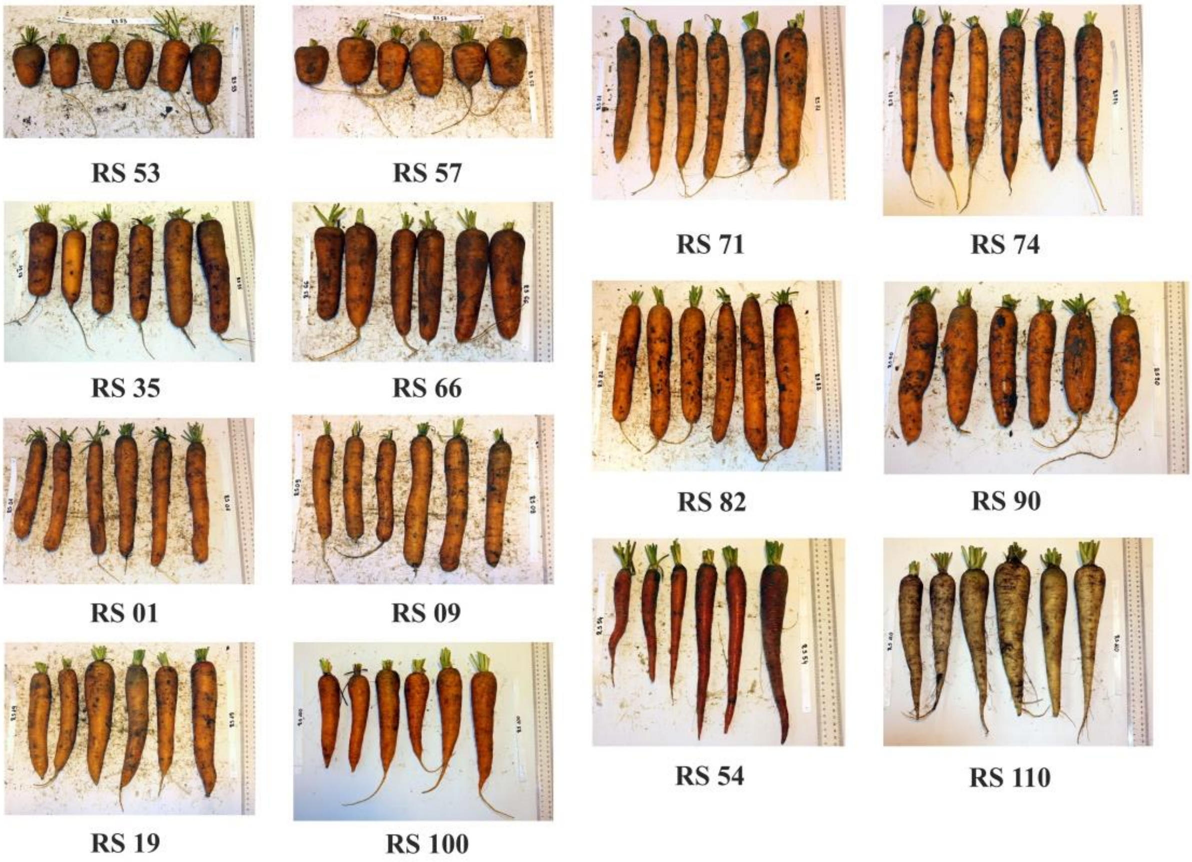

Supplementary figure 1. Examples of inter-and intra-cultivar root shape diversity. 'RS' codes represent accessions, as shown in Supplementary table 6.

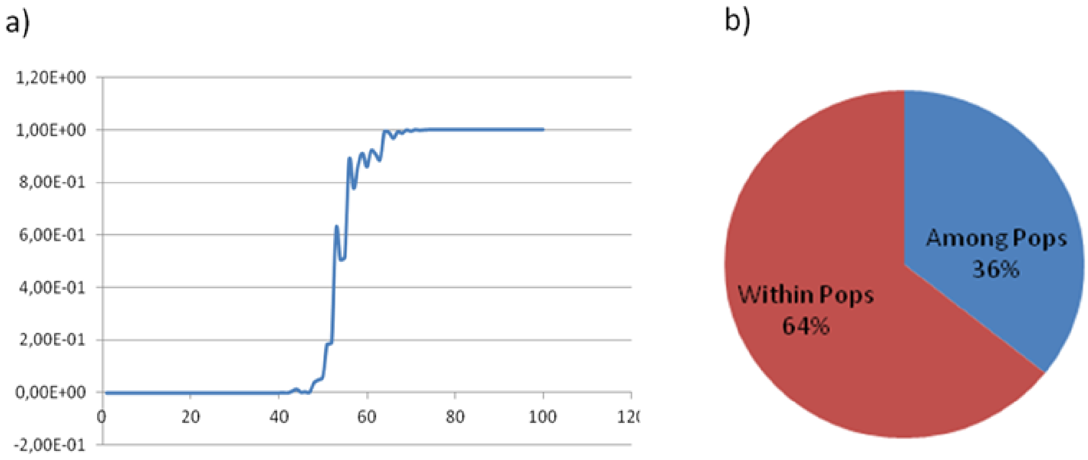

Supplementary figure 2. Plot representing significance (p-values) of first 100 PCA components based on the Tracy-Widom statistics (a) and percentage of molecular variance that can be attributed to diversity within and among populations (cultivars) (b).

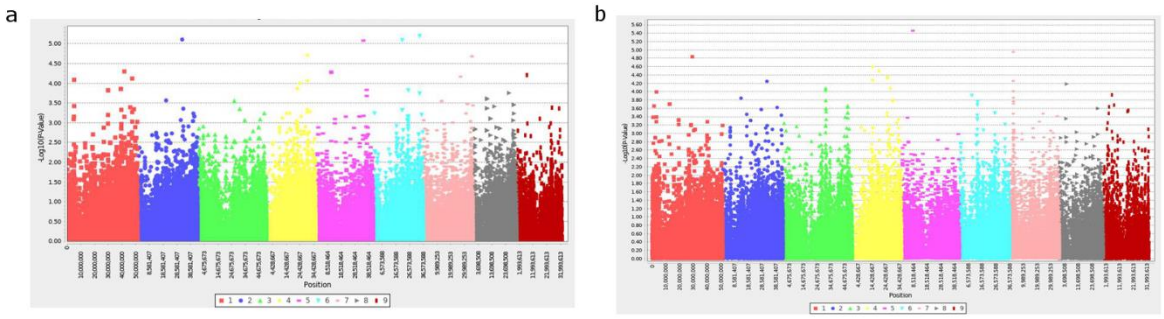

Supplementary Figure 3. Manhattan plot of association statistics for the carrot root collar (a) and length (b), showing SNP negative $\log_{10}$ p-values (y-axis) plotted against SNP positions on the nine carrot chromosomes (x-axis).

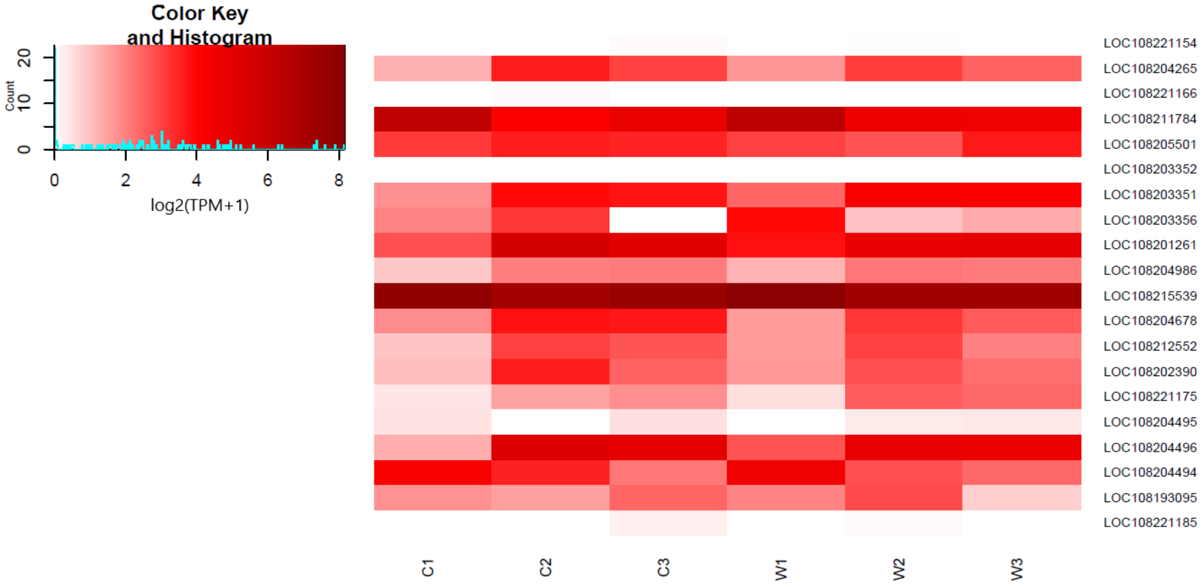

Supplementary Figure 4. The heat map of expression patterns of the 20 genes localized within the genomic region on chromosome 1 significantly associated with the shoulder diameter, analyzed for cultivated (C) and wild (W) carrot roots at different developmental stages (1-3), based on the previously published transcriptomic data [14]. The color scale represents $\log_2$(TPM+1) values.

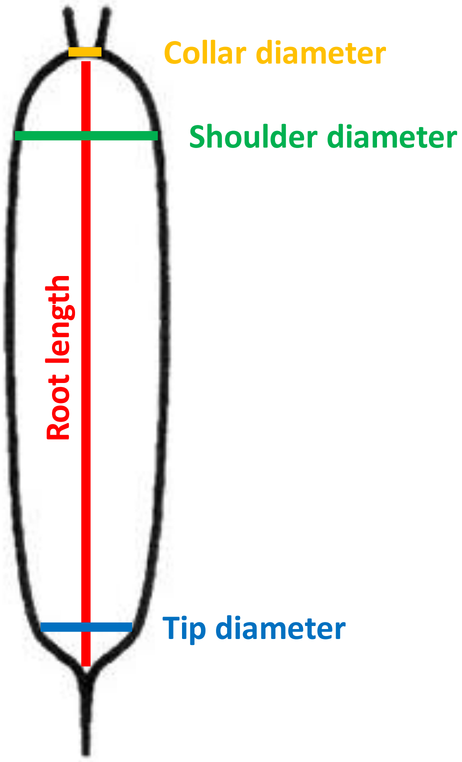

Supplementary Figure 5. Illustration of carrot storage root measurements
